# Host-bacteria metabolic crosstalk drives *S. aureus* biofilm

**DOI:** 10.15698/mic2021.05.749

**Published:** 2021-04-19

**Authors:** Kira L. Tomlinson, Sebastián A. Riquelme

**Affiliations:** 1 Department of Pediatrics, Columbia University, New York, NY 10032, USA.

**Keywords:** Staphylococcus aureus, biofilm, pneumonia, itaconate, immunometabolism

## Abstract

*Staphylococcus aureus* is a prominent pathogen that can cause intractable lung infections in humans. *S. aureus* persists in the airway despite inflammation and immune cell recruitment by adapting to host-derived antimicrobial factors. A key component of the immune response to infection are host metabolites that regulate inflammation and bacterial survival. In our recent paper (Tomlinson *et al.*, Nat Commun, doi: 10.1038/s41467-021-21718-y), we demonstrated that *S. aureus* induces the production of the immunoregulatory metabolite itaconate in airway immune cells by stimulating mitochondrial oxidant stress. Itaconate in turn inhibited *S. aureus* glycolysis and growth, and promoted carbon flux through bacterial metabolic pathways that support biofilm production. These itaconate-induced metabolic changes were recapitulated in a longitudinal series of clinical isolates from a patient with chronic staphylococcal lung infections, demonstrating a role for host immunometabolism in driving bacterial persistence during long-term staphylococcal lung infections.

Biofilms enable persistent staphylococcal infections in numerous tissues, including the bones, heart, lungs, and indwelling or implanted medical devices. These sessile communities of bacteria are encased in an extracellular matrix of proteins, nucleic acids, and polysaccharides that protect them from host phagocytes, oxidative stress, and antibiotics. Despite their importance in driving intractable infections, the factors that promote biofilm formation *in vivo* have not been well defined. Our study demonstrates that the host-derived metabolite itaconate drives staphylococcal biofilm formation by altering bacterial metabolism, reducing carbon flux through pathways used during planktonic proliferation like glycolysis and the tricarboxylic acid (TCA) cycle, and promoting pathways that support extracellular polysaccharide synthesis (**Figure 1**). Importantly, our studies focused on the role of itaconate in the host response to *S. aureus* in the lung, but itaconate is produced by myeloid cells that can respond to infection in most tissues. Further studies are warranted to determine if itaconate plays a role in the pathogenesis of persistent staphylococcal infections in other tissues.

**Figure 1:**
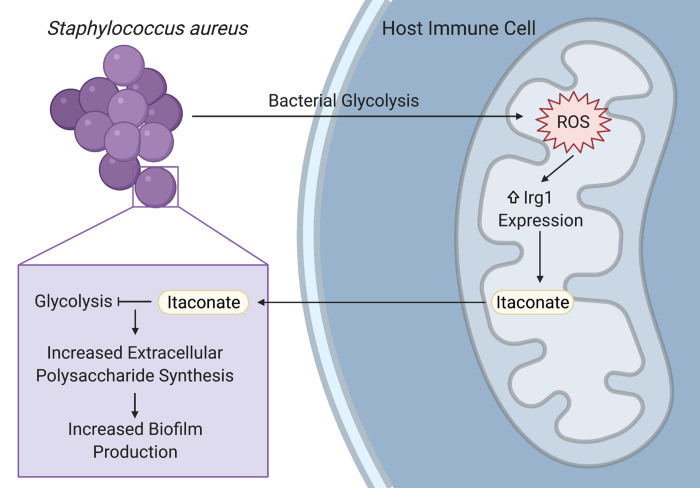
FIGURE 1: Summary of the host-pathogen metabolic crosstalk that drives *S. aureus* biofilms. *S. aureus* glycolysis induces mitochondrial oxidant stress in host immune cells, leading to increased *Irg1* expression and itaconate production. Itaconate inhibits *S. aureus* glycolysis and promotes the synthesis of extracellular polysaccharides used in biofilms. Created with Biorender.

Itaconate has been studied for its role in the immune response to lipopolysaccharide (LPS) and in the host-pathogen interactions that underlie Gram-negative infections. In these infections, itaconate can act as an antimicrobial by inhibiting the glyoxylate shunt, which enables bacteria to generate energy from carboxylic carbon sources without generating excess oxidant stress through the TCA cycle. Our study demonstrates that itaconate is also able to impose metabolic stress on *S. aureus*, which does not use the glyoxylate shunt, by directly inhibiting bacterial glycolysis (**Figure 1**). Specific opportunists have evolved itaconate degradation clusters that not only eliminate the toxic metabolite, but also enable the bacteria to use it as a carbon source. While *S. aureus* is not able to degrade or consume itaconate, another important lung pathogen, *Pseudomonas aeruginosa*, uses its itaconate degradation cluster to assimilate itaconate during biofilm production. Itaconate thus promotes bacterial biofilms through a variety of mechanisms, mirroring its promiscuity in mammalian cells and emphasizing the need for studies that will more broadly define its bacterial targets.

While previous studies mostly used LPS or Gram-negative bacteria to stimulate itaconate production, we demonstrated that itaconate is also produced in response to the Gram-positive *S. aureus*. Staphylococcal glycolysis induced mitochondrial oxidant stress in host immune cells, which lead to the upregulation of the itaconate synthetic enzyme, immune-responsive gene 1 (*Irg1*; **Figure 1**). Increased *Irg1* expression and itaconate production in response to mitochondrial oxidant stress may be a common mechanism between the Gram-positive and Gram-negative infections, given that the exact mechanism of itaconate induction has not been defined and that LPS also induces mitochondrial oxidant stress. This role for staphylococcal glycolysis in stimulating the host immune response in the airway mirrors its ability to activate proinflammatory cytokine production in other niches, like keratinocytes during skin infection. While the exact ligand interaction that links staphylococcal glycolysis to host cell activation still needs to be defined, these studies highlight the importance of studying bacterial metabolism in addition to other virulence factors when investigating the pathogenesis of infection.

Overall, our recent study defines a role for host-immunometabolism in driving bacterial persistence during staphylococcal lung infections. The clinical relevance of this metabolic crosstalk between host and pathogen is becoming increasingly apparent as numerous studies have defined metabolic interactions that underlie common infections. However, many questions remain and further investigation is needed to determine if these mechanisms play a role in the pathogenesis of infections at other tissue sites and with different pathogens.

